# Distribution and clinical significance of circulating CD8^+^CD28^−^ regulatory T cells in the peripheral blood of patients with pulmonary tuberculosis

**DOI:** 10.1186/s12890-022-02088-7

**Published:** 2022-07-30

**Authors:** Xin Yu, Yao Lin, Hui Chen, Min-Juan Wu, Li-Na Huang, Yi-Yan Song, Bin-Bin Gu, Zhi-Jian Ye, Ping Xu, Jian-Ping Zhang, Jun-Chi Xu

**Affiliations:** 1 Department of Clinical Laboratory, The Fifth People’s Hospital of Suzhou, 10, Guanqian Road, Suzhou, 215000 Jiangsu People’s Republic of China; 2 Department of Respiratory, The Affiliation Infections Diseases Hospital of Soochow University, 10 Guangqian Road, Suzhou, 215000 Jiangsu People’s Republic of China

**Keywords:** CD8^+^CD28^−^ Treg, Tuberculosis, Immune response

## Abstract

**Background:**

Regulatory T cells (Treg cells) in the peripheral blood of patients with pulmonary tuberculosis (PTB) may be closely related to the progression of PTB. In this study, the distribution characteristics and clinical importance of CD8^+^CD28^−^ Treg cells in patients with tuberculosis were systematically analyzed, and the role and importance of CD8^+^CD28^−^ Treg cells in influencing the immune response and progression of tuberculosis were discussed, which will provide immunological indices and reference values for the clinical diagnosis of tuberculosis.

**Methods:**

Flow cytometry, sputum smears and computed tomography imaging were used to analyze the distribution characteristics of CD8^+^CD28^−^ Treg cells in the peripheral blood of patients with PTB and the correlation between CD8^+^CD28^−^Treg cells and clinical and immune indices.

**Results:**

The percentages of CD4^+^CD25^high^ and CD8^+^CD28^−^ Treg cells in the peripheral blood of patients with PTB were significantly higher than those in the healthy control (HC) group. Further analysis showed that the percentage of CD4^+^CD25^high^Treg cells in the Stage II group was significantly higher than that in the HC group. The percentages of CD4^+^CD25^high^ and CD8^+^CD28^−^ Treg cells increased significantly in patients in the Stage II group. The proportion of CD8^+^CD28^−^ Treg cells was directly proportional to the degree of positivity in sputum smears, while CD4^+^CD25^high^Treg cells did not exhibit this trend.

The correlations between the percentage of CD4^+^CD25^high^ and CD8^+^CD28^−^ Treg cells and the percentage of lymphocyte subsets were examined. The percentage of CD8^+^CD28^−^ Treg cells was negatively correlated with the percentage of CD4^+^T cells and positively correlated with the CD8^+^T cell percentage in the HC and PTB groups. The percentage of CD4 + CD25^high^Treg cells was positively correlated with the percentage of CD4^+^T cells only in the PTB group.

**Conclusions:**

This study was the first to show that the proportion of CD8^+^CD28^−^ Treg cells in the peripheral blood of patients with PTB was significantly increased, and the increase in CD8^+^CD28^−^ Treg cells was related to the progression of PTB, which may affect the proportion of immune cell subsets by inhibiting the immune response, resulting in the progression of PTB.

**Supplementary Information:**

The online version contains supplementary material available at 10.1186/s12890-022-02088-7.

## Background

Tuberculosis (TB) is a chronic respiratory infectious disease caused by *Mycobacterium tuberculosis* (MTB), which seriously threatens human health and is the 13th leading cause of death worldwide. According to the 2021 Global Tuberculosis Report, there are approximately 9.9 million new TB patients worldwide, with 1.28 million deaths and a mortality rate of 17/100,000. Furthermore, due to the effect of the COVID-19 pandemic, the number of deaths due to TB in 2021 was higher than that observed in the previous year [1].

After MTB infection, cell-mediated immunity (CMI) and delayed type hypersensitivity (DTH) play important roles in the occurrence, development and prognosis of TB. CMI refers to the presence of a large number of activated macrophages produced by amplified levels of T lymphocytes and cytokines specific to MTB antigens, including those involved in the innate immune response and acquired immune response. Multiple immune cell subsets, their cytokines and specific TB-presenting cells are involved in the immune response and immune regulation of TB infection.

In recent years, the abnormal increase in the number of regulatory T cells (Treg cells) and the occurrence and development of immune responses and related diseases have attracted increasing attention. Studies have shown that the increase in the number of CD4^+^CD25^high^Treg cells in patients with pulmonary tuberculosis (PTB) may be related to low cellular immunity and lead to prolonged and repeated attacks of PTB [2]. When Treg cells are inhibited, anti-MTB immunity can return to normal, so Treg cells may be an important factor in the immune escape of TB. Treg cells play an immunosuppressive role mainly through cytokines and cell contact. Treg cells participate in the occurrence and development of diseases by inhibiting the immune response to self or foreign antigens. Many subsets of Treg cells have been identified, including CD4^+^CD25^high^Treg cells and CD8^+^CD28^−^Treg cells [3].

CD8^+^CD28^−^Treg cells are CD8^+^T cells lacking the CD28 molecule. CD8^+^CD28^−^Treg cells play a negative regulatory role in the activation of T cells and play an important regulatory role in controlling autoimmune diseases and rejection after organ transplantation. Recent studies have confirmed that CD8^+^CD28^−^Treg cells are an important subset that have the same immunosuppressive effects as CD4^+^CD25^high^Treg cells. CD8^+^CD28^−^Treg cells can upregulate the expression of IL-3, IL-4 and other inhibitory factors on the surface of antigen-presenting cells (APCs), which makes these cells immunologically tolerant; downregulates the expression of CD80, CD86 and other costimulatory molecules; and inhibits the activity of Th cells [4–7]. The transcription factor Foxp3 regulates the immunosuppressive effects of CD8^+^ Treg cells. The higher the expression levels of Foxp3, the stronger the inhibitory activity of CD8^+^ Treg cells, indicating a positive correlation. The ratio of CD8^+^CD28^−^Treg cells in the peripheral blood and tumor tissues of patients with primary liver cancer and gastric cancer was found to be significantly higher than that in normal individuals [8, 9]. In tumors, it was found that the number and activity of CD8^+^CD28^−^Treg cells in peripheral blood increased, which inhibited the antitumor immune response and reduced the antigen presentation ability of dendritic cells (DCs), ultimately leading to the onset of tumors [10–12]. In a study of HIV, the number of CD8^+^CD28^−^Treg cells was significantly increased and negatively correlated with the number of CD4^+^T cells, while in a study of pneumonia pathogens [13], CD8^+^CD28^−^Treg cells could be used to specifically diagnose *Mycoplasma pneumoniae* infection [14].

Because CD8^+^CD28^−^Treg cells exhibit increased secretion of IL-6 [15], which can inhibit the effects of IFN-γ and reduce the sensitivity of uninfected macrophages to IFN-γ stimulation, CD8^+^CD28^−^Treg cells may participate in the immune escape of MTB. However, there has been no report on the correlation between TB and CD8^+^CD28^−^Treg cells. Therefore, this study systematically analyzed the relationship between peripheral blood CD8^+^CD28^−^Treg cells and the course of TB and the immune response and further clarified the immune mechanism of TB.

## Methods

### Subjects and inclusion criteria

Subjects: From September 2018, to August 2019, 79 patients with PTB were recruited from the Tuberculosis Department of the Infectious Disease Hospital Affiliated with Soochow University. Computed Tomography (CT) imaging was combined with the clinical characteristics of patients, and the patients were divided into a severe PTB group (Stage II) and a mild PTB group (Stage I). In addition, according to the acid-fast staining results of sputum smears, the amount of mycobacteria excretion in PTB patients was measured and divided into four grades: + , +  + , +  +  + and +  +  +  +  + . Samples were collected from healthy controls (HCs) at the physical examination center of the fifth people’s hospital of Suzhou. The HCs were TB-IGRA negative, and no other respiratory tract infections or other diseases were confirmed in these participants.

Inclusion and exclusion criteria: The diagnosis of confirmed PTB met the diagnostic criteria for PTB, and patients were screened for sputum smears (+ and higher) and MTB cultures. CT imaging was combined with the clinical characteristics of patients to divide the patients into two groups: patients with lesions involving more than three lung fields; respiratory symptoms such as cough, expectoration and chest tightness; and a body temperature ≥ 39 °C were in the severe TB group (Stage II); in contrast, those with mild illness were in the mild group (Stage I). All cases of bacterial pneumonia were confirmed by clinical manifestations, etiological examinations and chest CT. Patients with other infectious diseases, including HIV, HCV, HDV and HBV infections, and other complications, such as diabetes, were excluded from the study.

### Reagents and instruments

Anti-human monoclonal antibodies (CD3-APC, CD4-PerCP CD25-FITC and CD28-PE) were purchased from BD Pharmingen Company (San Diego, CA, USA). Erythrocyte lysis buffer was purchased from BD Biosciences Company (San Diego, CA, USA). MTB drug-sensitive Roche medium (Roche, USA) was used.

Centrifuges (Jouan, France), an electronic balance (Eppendorff, Germany), a TB culture apparatus (BACTEC MGIT-960) and BBL MGIL culture tubes (BD Company, USA), a flow cytometer (BD Company, USA) and Philips MX4000 Dual Spiral CT Instrument (Philips Corporation, USA) were used.

### Detection of Treg cell subsets in human peripheral blood

CD25 was also considered to be a marker of CD4^+^Treg cells, and its expression level was correlated with Foxp3 and CD127 expression levels. (Additional file 1: Fig. S1), CD3^+^CD4^+^CD25^high^cells were considered to be CD4^+^Tregs in this study. Peripheral blood (100 μL) was collected from healthy individuals and PTB patients in each test group, and then antibodies (CD3-APC, CD4-PerCP CD25-FITC and CD28-PE) were added and incubated for 15 min. Then, red blood cells were lysed in erythrocyte lysis buffer, and the cells were centrifuged, washed with PBS, suspended in 0.5 mL of PBS, acquired by flow cytometry and analyzed by FlowJo software.

### Acid-fast staining of sputum smears

Sputum samples were examined by the acid-fast staining method, and the results of the microscopic examination were reported according to the following criteria: negative acid-fast bacilli ( − ) were observed continuously in 300 different visual fields, and no acid-fast bacilli were observed. The positive rate of acid-fast bacilli ( +) was 3–9 strips/100 visual fields. The positive rate of acid-fast bacilli (+ +) was 1–9 strips/10 visual fields. The positive rate of acid-fast bacilli (+ + +) was 1–9/field; the positive rate of acid-fast bacilli (+ +  +  + +) was ≥ 10 strips/field.

### Culture of MTB

The collected sputum samples were combined with an equal amount of 4% sodium hydroxide and mixed well. Five milliliters of the treated specimens was placed into a labeled sharp-bottomed centrifuge tube, shaken vigorously, and mixed well, after which PBS buffer was added, and the samples were centrifuged at 4000 rpm for 15 min. The supernatant was removed, and the pellet was resuspended in 12 mL of PBS. First, 0.8 mL of mixed additive (Growth Supplement) and miscellaneous bacterial inhibitor (PANTA) was added to the BBL MGIL culture tube, and 0.5 mL of the treated sample was then injected into the MGIT culture tube. The inoculated MGIT culture tube was placed into a BACTEC MGIT-960 TB culture instrument, and negative or positive culture results were obtained by automatic scanning by the instrument.

### Imaging and analysis of PTB patients

Patients with PTB were examined by spiral CT, and the imaging results were analyzed by two physicians who specialized in CT examination. The observation range was from the lung apex to the lung floor, and the CT imaging characteristics of PTB were recorded. CT imaging was combined with the clinical characteristics of patients to divide the patients into two groups: patients with lesions involving more than three lung fields; respiratory symptoms such as cough, expectoration and chest tightness; and a body temperature ≥ 39 °C in the severe TB group (Stage II). This group contained 1 patient with sputum smear + , 8 patients with sputum smear +  + , 12 patients with sputum smear +  +  + and 13 patients with sputum smear +  +  + . In contrast, those with mild illness were classified into the mild group (Stage I).

### Data sharing

The datasets generated and analyzed during the current study are available in the https://doi.org/10.6084/m9.figshare.19803757.v1 repository.

### Statistical analysis

The data were analyzed using the statistical software GraphPad Prism 6.0 (GraphPad Software, Inc., La Jolla, USA). The data are presented as the mean ± SEM. One-way ANOVA was used to perform comparisons, Pearson’s test was used to analyze correlations, and results with *p* values of < 0.05 were considered statistically significant.

## Results

### Basic clinical characteristics of selected subjects

The selected subjects were divided into the HC (control group) (n = 25) and PTB (n = 79) groups. Age, sex, TB severity, sputum smears and T lymphocyte subsets in the HC and PTB groups were analyzed (Table 1).

**Table 1:** Clinical characteristics of the study subjects

	HCs group	PTB group
Cases	43	98
Age (years)	42.58 ± 1.651	48.83 ± 1.951
Male/female	28/15	65/33
PTB progression(stage I/stage II)	NA	57/41
Bacillary load in sputum of PTB patients(+ / + + / + + + + / + + + +)	NA	19/25/26/28

Data are presented as number or mean ± SEM. PTB, Pulmonary tuberculosis; NA, not applicable

### Detection and analysis of CD8^+^CD28^−^ and CD4^+^CD25^high^Treg cells in the peripheral blood of PTB patients and HCs

**Fig. 1:**
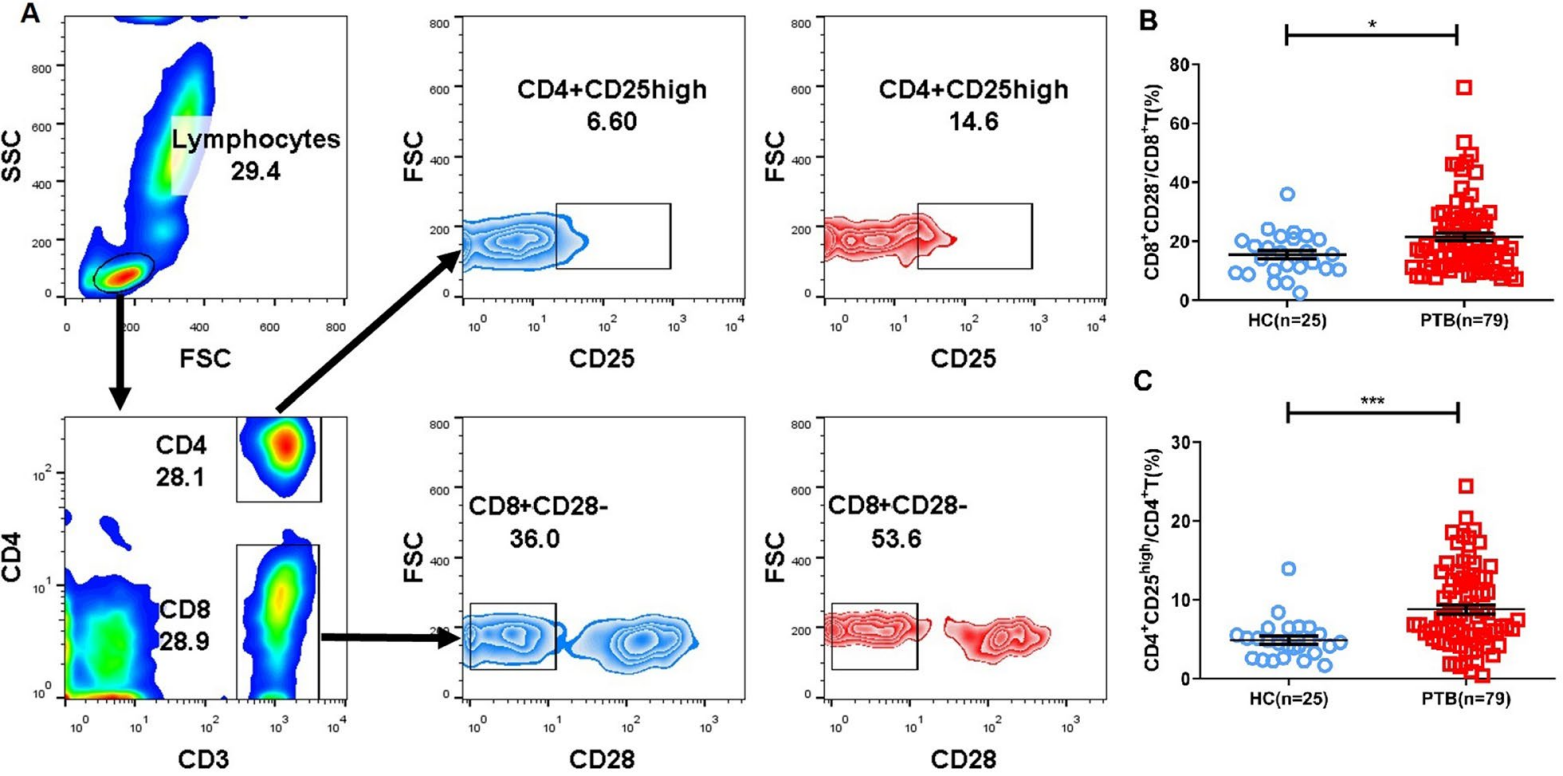
Gating strategy and frequencies of peripheral blood CD8^+^CD28^−^ and CD4^+^CD25^high^Treg cells in the HC group and PTB group. **A** The gating strategies and representative results showing the frequencies of peripheral blood CD8^+^CD28^−^ and CD4^+^CD25^high^Treg cells in the HC group and PTB group. **B** Statistical analysis of the frequencies of CD8^+^CD28^−^ and CD4^+^CD25^high^Treg cells in the HC and PTB groups

The percentages of CD4^+^CD25^high^ and CD8^+^CD28^−^Treg cells in the peripheral blood of subjects in the HC group and PTB group were analyzed by flow cytometry (Fig. 1A). The results showed that the percentages of CD8^+^CD28^−^Treg cells and CD4^+^CD25^high^Treg cells in the PTB group were significantly higher than those in the HC group (Fig. 1B, C).

### Analysis of the correlation between the number of peripheral blood CD8^+^CD28^−^ and CD4^+^CD25^high^ Treg cells and the course of TB

**Fig. 2:**
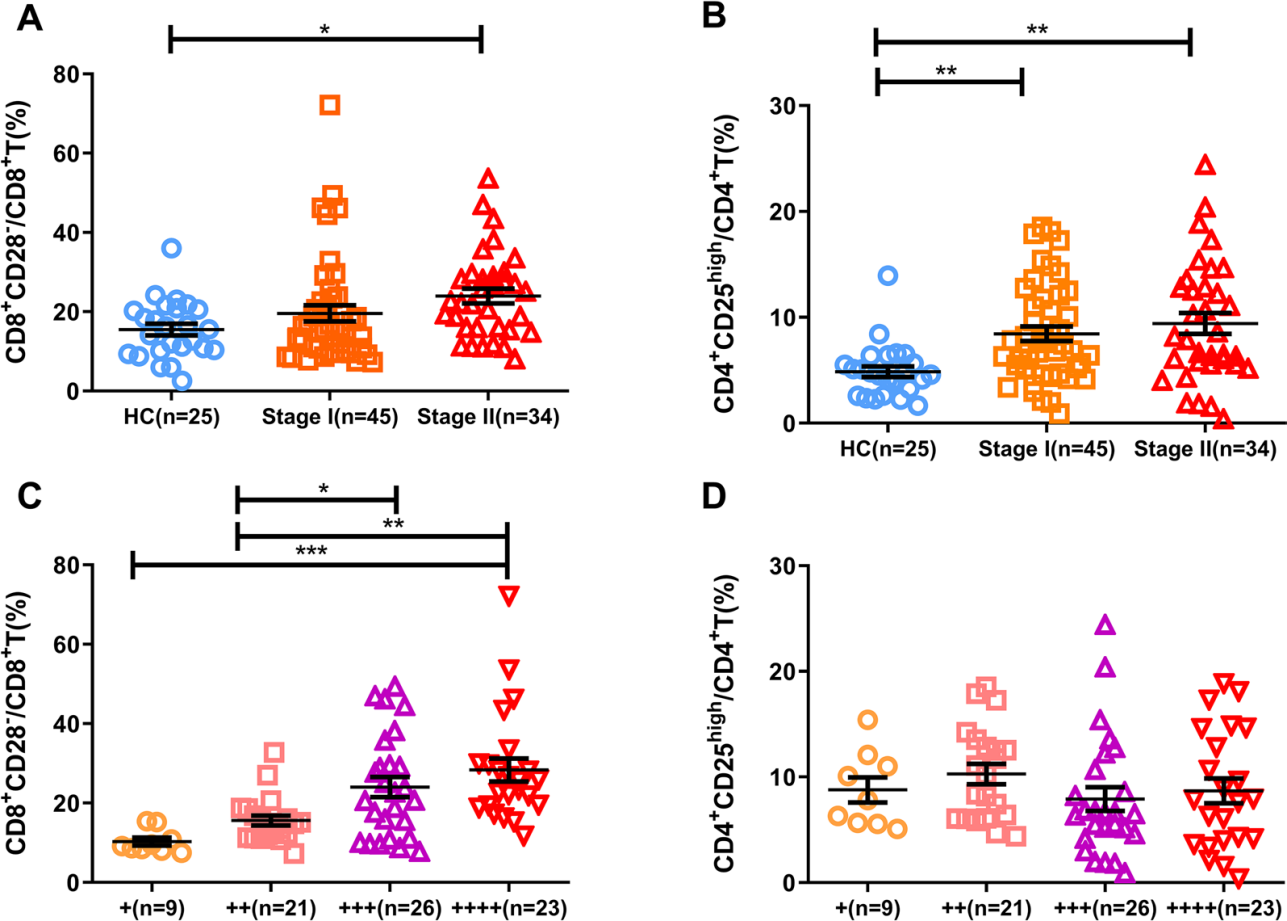
Correlation between the levels of peripheral blood CD8^+^CD28^−^ and CD4^+^CD25^high^Treg cells and the disease course of patients with TB. **A** Statistical analysis of the frequencies of CD8^+^CD28^−^Treg cells among the CD8^+^ population was compared among the HC, PTB-Stage I and PTB-Stage II groups. **B** Statistical analysis of the frequencies of CD4^+^CD25^high^Treg cells among the CD4^+^ population was compared among the HC, PTB-Stage I and PTB-Stage II groups. **C** Statistical analysis of the frequencies of CD8^+^CD28^−^ Treg cells among the CD8^+^ population was compared among the subgroups of PTB patients with different bacillary loads in their sputum. **D** Statistical analysis of the frequencies of CD4^+^CD25^high^Treg cells among the CD4^+^ population was compared among the subgroups of PTB patients with different bacillary loads in their sputum. + : 3–9 strips/100 visual fields, +  + : 1–9 strips/10 visual fields, +  +  + : 1–9/field and +  +  +  + : ≥ 10 strips/field

The proportions of CD8^+^CD28^−^ and CD4^+^CD25^high^Treg cells in stage I and stage II TB patients were compared with those in HCs, and the proportion of CD8^+^CD28^−^Treg cells in stage II patients was significantly higher than that in the HC group. The percentage of CD4^+^CD25^high^Treg cells in the Stage I and Stage II groups was higher than that in the HC group (Fig. 2A, B).

In addition, acid-fast staining of sputum smears was performed, and the amount of mycobacteria in PTB patients was used to divide the patients into four groups: + , +  + , +  +  + and +  +  + . The percentages of CD8^+^CD28^−^ and CD4^+^CD25^high^Treg cells were compared among patients in these four groups. The percentage of CD8^+^CD28^−^Treg cells was higher in the +  +  + group and +  +  +  + group than in the + group. The percentage of CD8^+^CD28^−^Treg cells in the +  +  +  + group was higher than that in the +  + group. However, there were no differences in the proportions of CD4^+^CD25^high^Treg cells (Fig. 2B).

### Analysis of the correlation between the levels of CD8^+^CD28^−^ and CD4^+^CD25^high^Treg cells and lymphocyte subsets in the peripheral blood of patients with TB

**Fig. 3:**
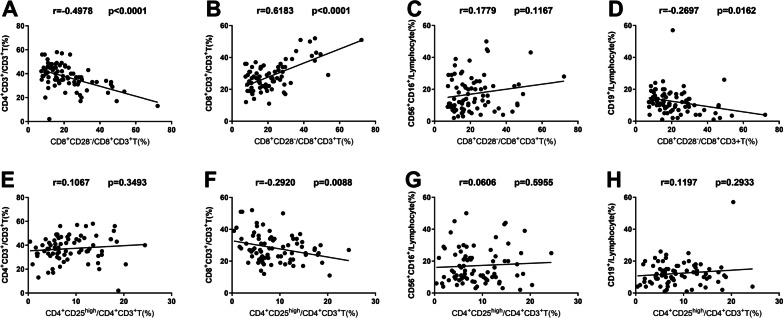
Correlation between the frequencies of peripheral blood CD8^+^CD28^−^ and CD4^+^CD25^high^Treg cells and lymphocyte subsets in the PTB groups. **A**–**D** Correlation between the frequencies of peripheral blood CD8^+^CD28^−^Treg cells and lymphocyte subsets in the PTB groups. **E**–**H** Correlation between the frequencies of peripheral blood CD4^+^CD25^high^Treg cells and lymphocyte subsets in the PTB groups

The percentage of peripheral blood CD8^**+**^CD28^**−**^Treg cells in patients with TB was correlated with the levels of lymphocyte subsets. The results showed that the proportion of CD8^**+**^CD28^−^Treg cells was negatively correlated with the proportion of CD4^+^T cells in the PTB group but was positively correlated with the CD8^+^T-cell percentage in the PTB group and HC group (Fig. 3A–D and Additional file 1: Fig. S2 A–D). There were no relationships between the proportions of CD4^+^CD25^high^ Treg cells and the levels of lymphocyte subsets in the PTB group and HCs (Fig. 3E–H and Additional file 1: Fig. S2 E–H).

## Discussion

Immunotherapy has been successful as a treatment for many diseases. Therefore, understanding the escape mechanism of TB and developing therapeutic methods for immune targets will provide ideas for the treatment of MDR-TB. At present, the immune escape mechanism of MTB has not been fully clarified, but it is thought that TB immunity is associated with type IV allergies and cellular immunity [16, 17]. With progress in TB immune research, an increasing number of scholars have found that the pathogenesis of TB is closely related to alveolar macrophages (AMs), T lymphocytes and related cytokines in vivo [18, 19]. Treg cells are important in maintaining immune balance and play important roles in autoimmune diseases, organ transplantation, bacterial and viral infections and the pathological states of various tumors [20–23].

An abnormal increase in the levels of CD4^+^CD25^high^Treg cells and the inhibition of the immune response to TB are important factors that cause low cellular immunity in patients with PTB and lead to prolonged and repeated attacks of PTB [2]. It has been shown that the number of CD4^+^CD25^high^Treg cells in TB patients is abnormally increased, which affects the duration and development of this disease by inhibiting effector immune cells [2]. In this study, the percentage of CD4^+^CD25^high^Treg cells in patients with PTB was examined and compared with that in healthy individuals, and the correlations between the levels of CD4^+^CD25^high^Treg cells, the levels of lymphocyte subsets and monocyte percentages were analyzed. The results showed that the percentage of CD4^+^CD25^high^Treg cells in the peripheral blood of PTB patients was higher than that in healthy individuals, suggesting that the initiation and continuation of the immune response amplified the number of CD4^+^CD25^high^Treg cells. As the body failed to clear MTB in time, the increase in the number of CD4^+^CD25^high^Treg cells further inhibited the effective anti-MTB immune response, resulting in an inadequate or insufficient response and leading to MTB escape and chronic infection. The results showed that there was no significant difference in the percentage of CD4^+^CD25^high^Treg cells among PTB patients with different grades of MTB, which indicated that the intensity of the MTB antigen and the deterioration of lung lesions had no significant effect on the number of CD4^+^CD25^high^Treg cells.

CD8^+^CD28^−^Treg cells are an important subgroup of Treg cells. Studies have shown that the number of CD8^+^CD28^−^Treg cells is increased in tumor patients, and these cells participate in the inhibition of the tumor-specific immune response and are closely related to tumor progression [3, 8]. John S Manavalan et al. [24] found the CD8^+^CD28^−^Tregs were Foxp3 positive in vitro and our study also found most CD8^+^Foxp3^+^T cells were CD28 negative (Additional file 1: Fig. S3), which prompt the CD8^+^CD28^−^Tregs have relationship with TGF-β and IL-10. In a study of viral pneumonia, it was found that CD8^+^Treg cells inhibited the function of CD8^+^T cells through IL-10 [25]. Another study found that CD8^+^CD28^−^Treg cells highly expressed type II immune response-related cytokines, such as IL-4, IL-10 and TGF-β [26]. The above studies suggest that CD8^+^CD28^−^Treg cells may play an important role in the formation of tuberculosis and granuloma. However, the distribution of these cells in PTB patients and the correlation with clinical symptoms and lymphocyte subsets are still unclear. Therefore, this study further examined the proportion of the CD8^+^CD28^−^Treg cell population in PTB patients compared with that in healthy individuals and analyzed the correlation between the numbers of these cells and lymphocyte subsets. The results showed that the percentage of CD8^+^CD28^−^Treg cells in the peripheral blood of PTB patients was higher than that in healthy individuals. In addition, the percentage of CD8^+^CD28^−^Treg cells in HCs and PTB patients correlated with the proportions of CD4^+^T cells and CD8^+^T cells, and this effect was different from that observed for the proportions of CD4^+^CD25^high^Treg cells, which was only associated with the proportions of CD8^+^T cells in PTB patients but not in HCs. This finding indicates that CD4^+^CD25^high^Treg cells belong to the induced cell population, and the proportion of induced cells increases with disease occurrence and progression and the initiation and continuation of the immune response, thus inhibiting the expansion of the immune response. As an intrinsic group of immune cells, CD8^+^CD28^−^Treg cells may regulate the proportions of each subgroup of immune cells. With the occurrence and progression of pneumonia, TB and other diseases and the initiation of the immune response, the proportion of these cells increases to limit an excessive immune response. However, in chronic infections such as TB, the abnormal increase in the number of these cells may inhibit an effective response. Further analysis showed that the percentage of CD8^+^CD28^−^Treg cells increased with the severity of PTB and the level of MTB excretion, which indicated that the intensity of MTB antigens and the degree of disease deterioration could affect the number of CD8^+^CD28^−^ Treg cells.

## Conclusions

In this study, the relationship between the CD8^+^CD28^−^Treg cell population and TB was analyzed systematically. The proportion of CD8^+^CD28^−^ cells in the peripheral blood of PTB patients was significantly increased, and there was a correlation between the proportion of CD8^+^CD28^−^Treg cells and the immune function of TB patients. Further examination showed that unlike the number of CD4^+^CD25^high^Treg cells, the increase in the number of CD8^+^CD28^−^Treg cells was related to the progression of PTB, which may affect the ratio of immune cell subsets by inhibiting the immune response, resulting in the progression of PTB. These results suggest that the CD8^+^CD28^−^Treg cell population may be an important immunosuppressive subgroup in TB patients and may become an important target of immune therapy for TB.

## Supplementary Information

**Additional file 1**. Analysis of the relationship between CD25, Foxp3 and CD127 on the CD4^+^ and CD8^+^ T cells.

## Data Availability

The datasets generated and analyzed during the current study are available in the https://doi.org/10.6084/m9.figshare.19803757.v1 repository.

## Abbreviations

PTB: Pulmonary tuberculosis
Treg cells: Regulatory T cells
HC: Healthy control
MTB: Mycobacterium tuberculosis
TB: Tuberculosis
CMI: Cell-mediated immunity
DTH: Delayed type hypersensitivity
DCs: Dendritic cells
AMs: Alveolar macrophages
CT: Computed tomography
