# Supplementary material for: Distribution and clinical significance of circulating CD8+CD28− regulatory T cells in the peripheral blood of patients with pulmonary tuberculosis
Source: BMC Pulm Med. 2022 Jul 30;22:291. doi: 10.1186/s12890-022-02088-7 (PMC9338636; doi:10.1186/s12890-022-02088-7)
Supplement: Supplementary file 1 — Additional file 1. Analysis of the relationship between CD25, Foxp3 and CD127 on the CD4+ and CD8+ T cells. [file 12890_2022_2088_MOESM1_ESM.docx]

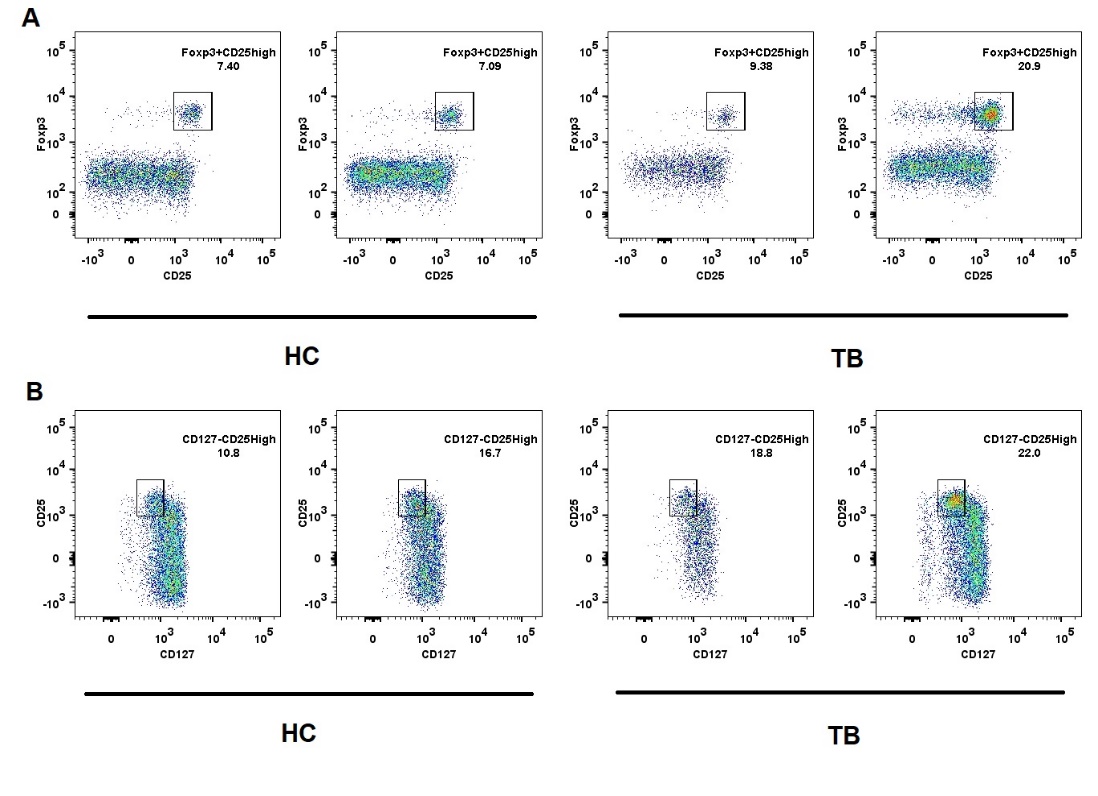


sFig. 1. Correlation between CD25 and Foxp3 and CD127 in CD4^+^T cells. (A) The relationship between CD25 and Foxp3. (B) The relationship between CD25 and CD127.


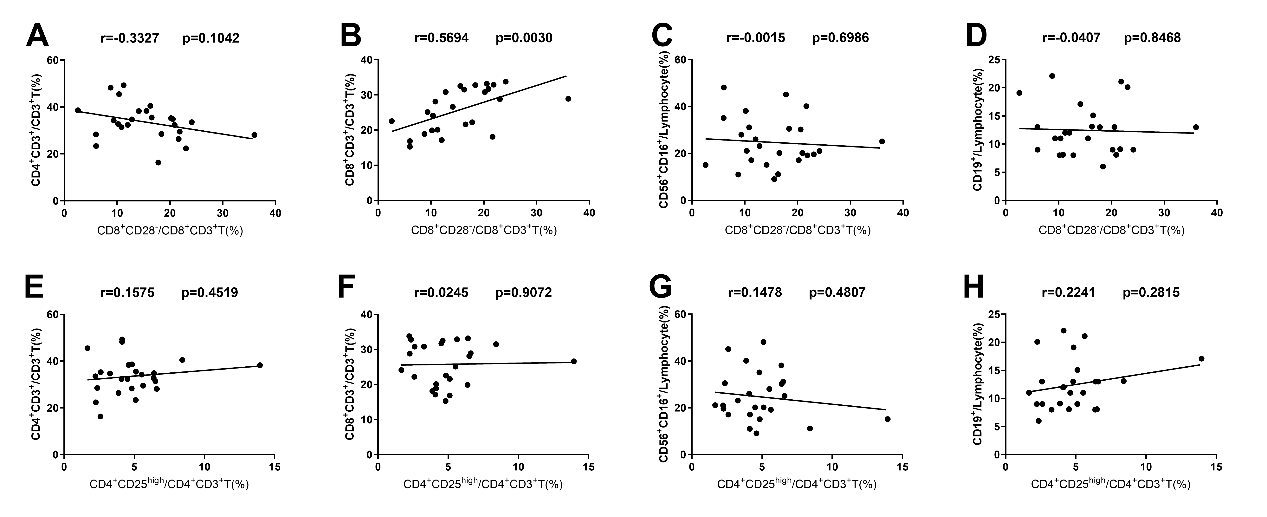


sFig. 2. Correlation between the frequencies of peripheral blood CD8^+^CD28^-^ and CD4^+^CD25^high^ Treg cells and lymphocyte subsets in the HCs groups. (A-D) Correlation between the frequencies of peripheral blood CD8^+^CD28^-^ Treg cells and lymphocyte subsets in the HCs groups. (E-H) Correlation between the frequencies of peripheral blood CD4^+^CD25^high^ Treg cells and lymphocyte subsets in the HCs groups.


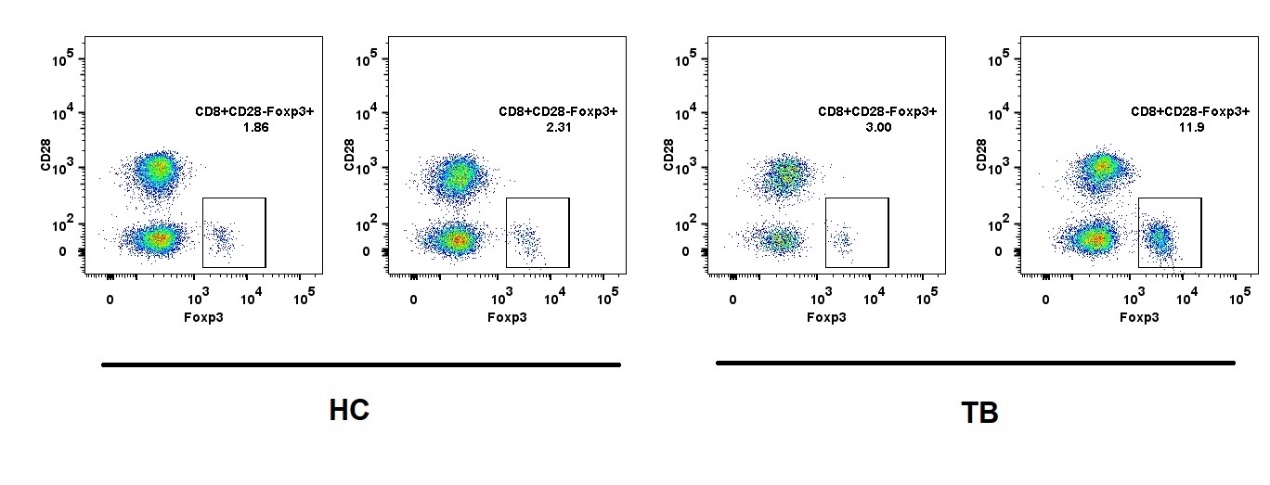
sFig. 3. The expression of Foxp3 in CD8+CD28-Treg cells.
